# Periconceptional changes in thyroid function: a longitudinal study

**DOI:** 10.1186/1477-7827-10-20

**Published:** 2012-03-21

**Authors:** Ursula Balthazar, Anne Z Steiner

**Affiliations:** 1 Anchorage Women's Clinic, Anchorage, AK, USA; 2 Department of Obstetrics and Gynecology, University of North Carolina at Chapel Hill, Chapel Hill, NC, USA

**Keywords:** Thyroid, Pregnancy, Conception

## Abstract

**Background:**

Limitations in our current knowledge of normative physiologic changes in thyroid function during the periconception window narrow our ability to establish an optimal approach to screening and diagnosis of thyroid disease in pregnant women. The objective of this study was to characterize changes in thyroid function during the transition from the pre-pregnant to pregnant state in normal fertile women.

**Methods:**

Women (N = 60) ages 30-42 years without a history of thyroid disease, who were planning pregnancy, were observed prospectively before and during early pregnancy. Thyroid function (thyroid stimulating hormone, TSH and free thyroxine, FT4) was measured before conception and between 6 and 9 weeks gestation. Pre-pregnancy samples were analyzed for thyroid antibodies. Bivariate analyses and longitudinal curves (general estimating equation models) were used to analyze changes in thyroid function during the periconception window by antibody status.

**Results:**

Pre-pregnancy TSH values were significantly higher than early pregnancy TSH (p < 0.001), but FT4 values did not differ (p = 0.53). TSH declined as gestational age increased (P < 0.01). Thyroid antibody positive women had a higher pre-pregnancy TSH compared to antibody negative women (p < 0.01). Periconceptional change in thyroid function was more variable among women with antibodies (p < 0.001). 50% of women with elevated pre-pregnancy TSH values (TSH > 3.0 mIU/L) had normal TSH values (TSH < 2.5 mIU/L) in pregnancy.

**Conclusions:**

TSH values decline during the transition from pre-pregnancy to early pregnancy. The change in TSH appears to be less predictable in women with thyroid antibodies. Periconceptional changes in thyroid function should be considered in formulating prenatal thyroid screening guidelines.

## Background

Reproductive hormones have been shown to impact thyroid physiology during pregnancy [1-3]. Estrogens stimulate thyroid binding globulin (TBG) production by the liver effectively decreasing free thyroxine (FT4) [1]. Human chorionic gonadotropin binds to and activates the thyroid stimulating hormone (TSH) receptor [2,3]. Therefore, high levels of reproductive hormones produced during pregnancy likely lead to alterations in maternal thyroid function and measures of thyroid function (TSH and FT4 levels). Normal maternal thyroid function during the periconception window defined as the transition from pre-pregnancy through the early first trimester is important as: 1) implantation disorders may predispose to adverse obstetrical outcomes [4,5], 2) most miscarriages occur during this interval [6,7], and 3) normal early fetal neurological development requires maternal thyroxine [8].

Currently, normative data defining thyroid function during early pregnancy derives primarily from cross-sectional population-based studies of pregnant women [9-11]. Use of cross-sectional data assumes 1) pregnancy induced changes in thyroid function are the same for all women, 2) women with normal pre-pregnancy thyroid levels will have normal pregnancy thyroid levels, and 3) values outside of 95% confidence limits for the population represent thyroid dysfunction. Longitudinal data relating to changes in thyroid function surrounding the time of conception are limited to studies in women with thyroid disease or infertility and cannot be extrapolated to a general population of healthy, fertile women [1,12,13].

Gaps in our current knowledge of normal physiologic changes in thyroid function during the periconception window limit our ability to establish effective methods for pre-conception screening and treatment and hamper efforts to identify potential "at risk" populations that may benefit from early intervention. We conducted a prospective observational study aimed at characterizing the periconception changes in thyroid function in normal fertile women with and without thyroid antibodies. We analyzed data derived from blood samples obtained before conception and during early viable pregnancies in a group of women who conceived without medical assistance in less than 6 months.

## Methods

### Study design

This prospective observational study included 60 women between the ages of 30 and 44 years. All subjects were participants in *Time to Conceive (TTC)*, an on-going study approved by the Institutional Review Board at the University of North Carolina at Chapel Hill [14]. *TTC *enrolls women 30-44 years of age with proven or untested fertility who have been actively attempting pregnancy for less than 3 months. A blood sample is obtained during the early follicular phase in the first menstrual cycle after enrollment and sera are stored at -80°C. In those who conceive, transvaginal ultrasonography is performed between 6 and 9 weeks gestation (based on last menstrual period). *TTC *study participants reporting no history of thyroid disease who conceived viable pregnancies between January 2009 and April 2010 were recruited to participate in this study; written consent was obtained during the scheduled visit for ultrasonography. The early pregnancy blood sample was obtained at the time of the viability ultrasound.

Blood samples obtained before and during early pregnancy were processed in the same fashion. Ultrasonography was performed by one of the two authors using standard technique and a vaginal probe (5 MHz). Gestational age was defined by the crown-rump length obtained during the ultrasound.

### Thyroid hormone assays

Pre-pregnancy samples were analyzed for thyroperoxidase (TPO) and thyroglobulin (TG) antibodies, TSH, and free thyroxine (FT4); those obtained during pregnancy were analyzed only for TSH and FT4. Serum samples were shipped on dry ice via overnight carrier to the University of Southern California Endocrinology Laboratories and all samples were analyzed at the same time. Serum TSH and FT4 were measured using a third generation electrochemiluminescent immunoassay (Roche Elecsys 2010 Analyzer); reference values were 0.30-3.0 mIU/liter for TSH and 0.80-2.00 ng/dL for FT4. The intra- and interassay variation coefficients were 4.0% and 3.6% for TSH and 2.4% and 4.0% for FT4. TPO and TG antibodies were measured using a radioimmunoassay kit (Kronus, Star, ID) with values greater than 1.0 U/mL considered positive; the intra- and inter-assay coefficients of variation were less than 9% for both assays.

### Statistical analysis

Descriptive statistics were calculated for TSH and FT4 for all subjects at baseline (preconception) and during early pregnancy (6-9 weeks gestation, based on crown-rump length) and in subjects with and without thyroid antibodies (TPO or TG). TSH was not normally distributed. For statistical analyses, TSH values were natural log (ln)-transformed to achieve normalcy and accommodate for outliers. Groups were compared using the student *t*-test. Preconception and pregnancy hormone levels were compared using a paired *t*-test. Geometric means are presented for TSH for comparison of groups. One sample test of proportion was used to compare the prevalence of thyroid antibody positive women to the prevalence in the general population.

To compare changes in hormone levels over the periconception window by presence of thyroid antibodies general estimating equation (GEE) models were created. These models allow one to analyze changes in TSH concentrations in subjects with and without thyroid antibodies, using a variable for gestational age (preconception set as 0) to analyze the independent effect of gestational age on TSH levels, using a variable for antibody status to analyze the independent effect of thyroid antibodies on TSH levels, and using an interaction term defined by the product of gestational age and antibody status to compare the pattern of change (or shape of the hormone curve) by antibody status. GEE models allow for analysis of data with repeated measurements on the same subject over time, differing from repeated measure analysis of variance (MANOVA) in that the method does not require an equal number of measurements and equal time spacing. Similar analyses were conducted with FT4. All analyses were conducted using STATA 11.0 (College Station, TX).

## Results

The median age of subjects was 33 years (range 30-43 years); 44/60 (73%) were under age 35; 51 (86%) were White, 4 (6%) Black, 4 (6%) Asian, and 1 (2%) had mixed race/ethnicity (Table 1). The median preconception TSH was 2.0 mIU/L (Interquartile range (IQR) 1.3-2.45) and the corresponding FT4 was 1.22 ng/dL (IQR 1.15-1.26). The median gestational age at time of early pregnancy ultrasound was 7.4 weeks gestation (IQR 7.0-7.7). All pregnancies were singletons and were achieved in less than 6 menstrual cycles.

**Table 1:** Characteristics of the study population

	All women	Antibody positive women	Antibody negative women	TSH ≥ 3.0 mIU/liter
**Subjects**	60	16	44	8

**Age (years)^a^**	33 (30-34)	31.5 (30-33)	33 (31-36)	30.5 (30-32)

**Ethnicity**				

**White**	52 (86%)	13 (81%)	39 (89%)	7 (88%)

**African American**	4 (6%)	2 (13%)	2 (5%)	1 (12%)

**Asian**	4 (6%)	0 (0%)	4 (9%)	0 (0%)

**Other**	1 (2%)	1 (6%)	0 (0%)	0 (0%)

**Preconception TSH (mIU/L)^b^**	1.9 ± 1.9	2.68 ± 2.13	1.63 ± 1.67	3.78 ± 1.24

**Preconception Free T4 (ng/dL)^c^**	1.22 ± 0.18	1.18 ± 0.16	1.23 ± 0.18	1.18 ± 0.20

**Gestational Age^a ^(weeks)**	7.4 (7.0-7.7)	7.3 (6.9-7.6)	7.4 (7.1-7.9)	6.6 (6.4-7.6)

**Early pregnancy TSH ^b ^(mIU/L)**	1.45 ± 2.2	2.13 ± 2.96	1.26 ± 2.00	2.79 ± 1.56

**Early pregnancy Free T4 (ng/dL)^c^**	1.23 ± 0.16	1.20 ± 0.16	1.24 ± 0.16	1.22 ± 0.09

^a ^Median (Interquartile range)

^b ^Geometric mean ± standard deviation

^c ^Mean ± standard deviation

**Figure 1:**
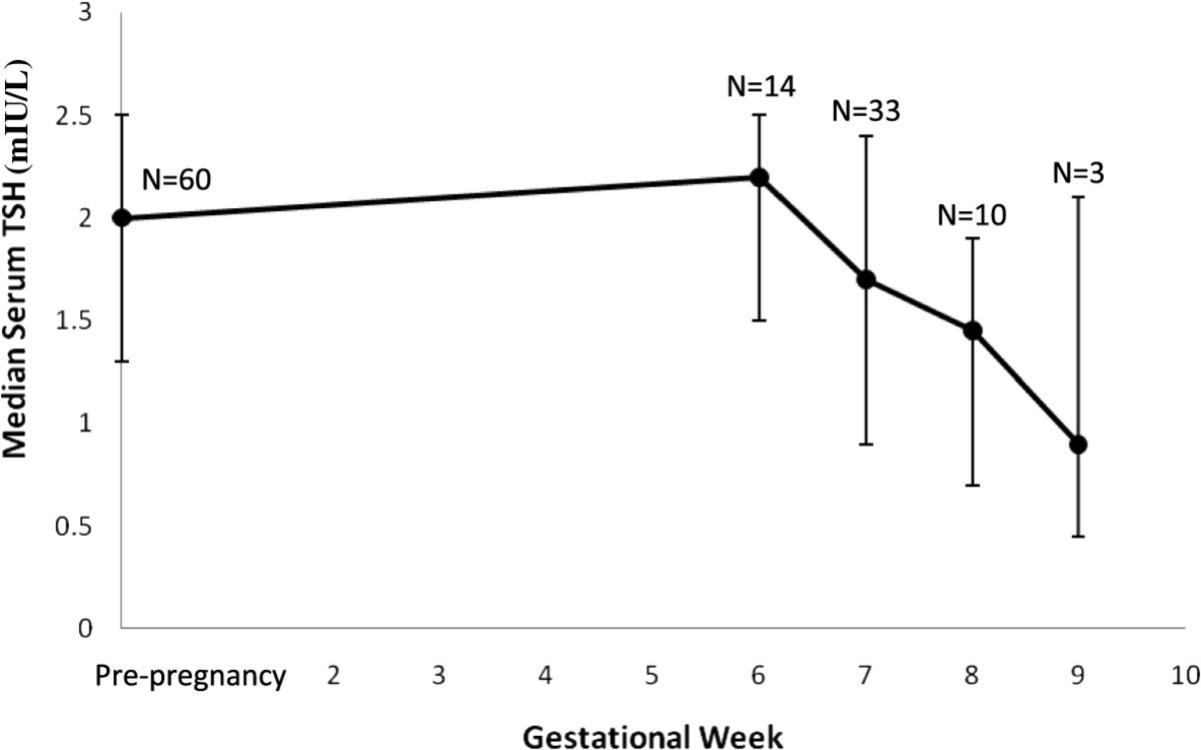
**Periconception changes in TSH**. Median (with interquartile range) serum TSH concentrations (mIU/L) are shown in the 60 study participants across the preconception and early pregnancy time frame defined by our study (< 10 weeks). Pre-pregnancy values are presented at 0 weeks gestation.

In the study cohort, the early pregnancy TSH values were significantly lower than the preconception TSH values (geometric mean ± standard deviation (SD): 1.45 ± 2.2 vs. 1.9 ± 1.9 mIU/L, p < 0.001). The pattern of change in median TSH levels across the periconceptional interval and early pregnancy (< 10 weeks) is illustrated in Figure 1. TSH values before pregnancy and at 6 weeks gestation were similar. After 6 weeks gestation, TSH values declined significantly as gestational age increased (P_gestational age _< 0.01). FT4 levels remained unchanged over the periconception window (mean ± SD: pre-pregnancy 1.22 ± 0.18 vs. pregnancy 1.23 ± 0.16 ng/dL, p = 0.5).

Sixteen of the 60 subjects (26.6%) had detectable thyroid antibodies; 10 (17%) had TPO antibodies, 13 (22%) had TG antibodies, and 7 (12%) had both. The preconception TSH levels were significantly higher in women with antibodies than in those without antibodies (geometric mean ± SD 2.68 ± 2.13 vs. 1.63 ± 1.67 mIU/L, p = 0.005); pre-pregnancy FT4 values were not different (Table 1). An analysis of the change in TSH levels during periconception by presence of antibodies revealed that TSH levels in women with antibodies were significantly higher than in those without antibodies (P_antibody presence _< 0.001); the overall pattern of change was similar in the two groups (P_antibody presence * gestational age _= 0.96).

The mean change in TSH from preconception to early pregnancy was -0.40 mIU/L (range -7.2 to 2.4). Mean change in TSH did not statistically differ by antibody status (antibody positive group -0.65 ± 2.2 mIU/L; antibody negative group -0.31 ± 0.60 mIU/L, p = 0.36); however, the change in TSH was more variable among women with antibodies (variance ratio test, P < 0.001).

We further analyzed our data in the subset of eight subjects (13%) having a preconception TSH concentration greater than 3.0 mIU/L (> 2 SD above the mean established in the laboratory), all having a normal pre-pregnancy FT4 levels (Table 1). In this subgroup, preconception TSH values were again significantly higher than TSH values observed during early pregnancy (geometric mean ± SD 3.78 ± 1.24 vs. 2.79 ± 1.56 mIU/L, p < 0.001); 4/8 (50%) had a normal early pregnancy TSH concentration (< 2.5 mIU/L).

## Discussion

Our unique longitudinal design allowed us to examine periconception changes in thyroid function within a group of women with no known history of thyroid disease or infertility. Longitudinal data confirmed previous cross-sectional data that implied that a woman's TSH values would decline from pre-pregnancy to early pregnancy. TSH started to decline after 6 weeks gestation. Presence of thyroid antibodies was associated with higher pre-pregnancy and early pregnancy TSH values. While absolute change in TSH did not differ significantly between women with and without thyroid antibodies, change in TSH was more variable among women with antibodies. Fifty percent of women with abnormal pre-pregnancy TSH values had TSH values within the normal range in early pregnancy.

Our results are consistent with those of previous large population-based studies observing that TSH levels decline gradually as the concentration of HCG increases during early pregnancy [9,10]. While these studies described women following the detection of pregnancy, our study focuses on the transition from the non-pregnant to pregnant state. Although FT4 concentrations during the periconceptional interval were unchanged, TSH is arguably the more sensitive and accurate measure of the tissue levels and activity of thyroxine.

Our observation that detectable thyroid antibodies were associated with a higher median preconception TSH concentration is consistent with previous reports that women with thyroid antibodies generally exhibit higher serum TSH levels before and during pregnancy [2,15,16]. However, these previous studies included infertile women or women with pre-existing hypothyroidism, conditions which themselves may have altered thyroid function and pregnancy outcomes. Unlike the previous studies, our subjects were not infertile and did not have a previous diagnosis of hypothyroidism.

Our data suggest that the higher TSH concentrations observed before and during early pregnancy in women with thyroid antibodies more likely reflects a possible subclinical pathology. Even with TSH and FT4 levels within the "normal" range prior to pregnancy, antibody positive women may have compromised basal thyroid function. Among women with thyroid antibodies, the absolute change in TSH with pregnancy was highly variable, implying that a pre-pregnancy TSH value will not necessarily predict pregnancy thyroid function among women with antibodies. Pregnancy may serve as a provocative test of thyroid status among women with antibodies. Unfortunately, for a subgroup, pregnancy may lead to thyroid dysfunction, placing them at increased risk for poor pregnancy outcomes including miscarriage [15,17].

Our data raise questions regarding current thyroid screening guidelines, which recommend targeted screening for thyroid disease in high-risk groups during pregnancy [18,19]. The majority of women typically do not begin prenatal care before 8-10 weeks gestation, when TSH concentrations already have decreased significantly from preconception levels. Our data suggest that TSH concentrations start declining after 6 weeks gestation. Consequently, patients with subclinical thyroid dysfunction (as determined by elevated pre-pregnancy TSH values) may fail to be detected by thyroid screening in the first trimester. This discrepancy may explain the lack of a consistent association between pregnancy TSH values and miscarriage [20] despite an observed association between thyroid antibodies and miscarriage [21].

In our study cohort, eight (13%) subjects had TSH levels more than 2 SD above the mean before conception. Four of the eight (50%) had a normal TSH concentration in early pregnancy (< 2.5 mIU/liter) and would have screened normal if only tested antenatally. If preconception testing is the more accurate measure of maternal thyroid function, screening during pregnancy may yield falsely reassuring results unless thyroid antibody status is considered in the evaluation and treatment algorithm. Conversely, if testing during early pregnancy best reflects true maternal thyroid function, preconception screening may result in over-diagnosis of hypothyroidism and unnecessary treatment in a significant proportion of women. Conclusions from this observation are limited due our small sample size.

Our study is the first to examine longitudinal periconceptional changes in thyroid hormone levels in a normal fertile population. This study design allows us to understand the relationship between pre-pregnancy and pregnancy thyroid function within an individual. Knowledge gained from such studies can be used to determine the most appropriate timing for thyroid screening among reproductively competent women. The subjects enrolled had no prior history of thyroid disease and exhibited normal fecundity by having conceived within six menstrual cycles. Moreover, there were no differences in the median ages of subgroups with and without thyroid antibodies and those having a preconceptional TSH concentration greater than 3.0 mIU/L.

The proportion of women in our cohort having detectable thyroid antibodies (16/60, 27%) was somewhat higher than has been observed in larger populations (14-18%), but the difference is not statistically significant (p = 0.07) [11]. Our study used an assay with a lower antibody detection level (1 U/ml) than used in some previous studies of thyroid autoimmunity and pregnancy [9,17]. It is possible that the periconceptional changes in TSH may be more pronounced in women with higher thyroid antibody levels and in populations where antibody positivity is defined by a higher threshold, potentially increasing the likelihood for a negative reproductive outcome.

Our study has several limitations. The cohort of subjects was relatively small, including only 60 women. More importantly, the patterns of periconceptional change in measures of thyroid function were defined by sampling at only two time points in each individual. Nonetheless, our observations indicate the need for larger and more sophisticated longitudinal studies with serial testing immediately before conception and at frequent intervals during early pregnancy, ideally beginning at four weeks gestation, to better define the patterns of periconceptional change in measures of thyroid function and determine their clinical relevance. In addition, our sample was limited to women between the ages of 30-42 years and therefore may not accurately represent the larger population, but the narrower age range of the subjects in our study improved our ability to examine the impact of thyroid antibodies on periconceptional changes in thyroid function.

## Conclusions

The periconception interval encompasses dynamic changes in thyroid function, as reflected by a progressive decrease in serum TSH concentrations. Moreover, the patterns of change appear to be highly variable among women with thyroid antibodies. Current guidelines for screening thyroid function only during early pregnancy may fail to identify all women who might benefit from early intervention.

## Competing interests

The authors declare that they have no competing interests.

## Authors' contributions

Dr. AZS: conception and design, acquisition of data, analysis and interpretation; revising it critically for important intellectual content; has given final approval of the version to be published. Dr. UB: acquisition of data, analysis and interpretation of data; drafting of the manuscript; has given final approval of the version to be published. Both authors read and approved the final manuscript.
